# Review of the current empirical literature on using videoconferencing to deliver individual psychotherapies to adults with mental health problems

**DOI:** 10.1111/papt.12332

**Published:** 2021-02-23

**Authors:** Neil Thomas, Caity McDonald, Kathleen de Boer, Rachel M. Brand, Maja Nedeljkovic, Liz Seabrook

**Affiliations:** ^1^ National eTherapy Centre Swinburne University of Technology Melbourne Victoria Australia; ^2^ Centre for Mental Health Swinburne University of Technology Melbourne Victoria Australia; ^3^ Alfred Hospital Melbourne Victoria Australia; ^4^ School of Health and Behavioural Sciences University of the Sunshine Coast Sippy Downs Qld Australia

**Keywords:** telehealth, telemental health, videoconferencing psychotherapy, cognitive behavioural therapy

## Abstract

**Purpose:**

The COVID‐19 pandemic has resulted in a widespread adoption of videoconferencing as a communication medium in mental health service delivery. This review considers the empirical literature to date on using videoconferencing to deliver psychological therapy to adults presenting with mental health problems.

**Method:**

Papers were identified via search of relevant databases. Quantitative and qualitative data were extracted and synthesized on uptake, feasibility, outcomes, and participant and therapist experiences.

**Results:**

Videoconferencing has an established evidence base in the delivery of cognitive behavioural therapies for post‐traumatic stress disorder and depression, with prolonged exposure, cognitive processing therapy, and behavioural activation non‐inferior to in‐person delivery. There are large trials reporting efficacy for health anxiety and bulimia nervosa compared with treatment‐as‐usual. Initial studies show applicability of cognitive behavioural therapies for other anxiety and eating disorders and obsessive–compulsive spectrum disorders, but there has yet to be study of use in severe and complex mental health problems. Therapists may find it more difficult to judge non‐verbal behaviour, and there may be initial discomfort while adapting to videoconferencing, but client ratings of the therapeutic alliance are similar to in‐person therapy, and videoconferencing may have advantages such as being less confronting. There may be useful opportunities for videoconferencing in embedding therapy delivery within the client’s own environment.

**Conclusions:**

Videoconferencing is an accessible and effective modality for therapy delivery. Future research needs to extend beyond testing whether videoconferencing can replicate in‐person therapy delivery to consider unique therapeutic affordances of the videoconferencing modality.

**Practitioner points:**

- Videoconferencing is an efficacious means of delivering behavioural and cognitive therapies to adults with mental health problems.
- Trial evidence has established it is no less efficacious than in‐person therapy for prolonged exposure, cognitive processing therapy, and behavioural activation.
- While therapists report nonverbal feedback being harder to judge, and clients can take time to adapt to videoconferencing, clients rate the therapeutic alliance and satisfaction similarly to therapy in‐person.
- Videoconferencing provides opportunities to integrate therapeutic exercises within the person’s day‐to‐day environment.

## Background

One of the notable impacts of the COVID‐19 pandemic on psychological therapy delivery has been the use of videoconferencing becoming widespread. Telehealth—the use of telecommunication technology to deliver services—includes a range of networked communication modalities, also including telephone, email, and text chat messaging. With improvements in online videoconferencing software and internet speeds, products such as Skype, FaceTime, and Zoom had already entered mainstream personal and business communication usage prior to the pandemic, and by incorporating video offer a close approximation to being in‐person. While in‐person services had been slow to adopt digital technologies prior to the pandemic, substantial potential was already seen in making services more accessible, and the necessity to minimize in‐person interaction has catalysed the adoption of this technology in psychotherapy delivery (Chen et al., 2020; Shore, Schneck, & Mishkind, 2020; Torous, Myrick, Rauseo‐Ricupero, & Firth, 2020; Wind, Rijkeboer, Andersson, & Riper, 2020).

Videoconferencing has been researched as a medium for therapy delivery over the past three decades, adopting contemporaneous communication technologies such as television‐based telemedicine equipment, videophones, and Internet‐based webcam systems (Simpson, 2009). While therapists report concerns about technical difficulties and the potential impact of videoconferencing on the therapeutic alliance (Connoly, Miller, Lindsay, & Bauer, 2020; Simpson & Reid, 2014), they make adaptations such as emphasizing their own non‐verbal behaviour and clarifying the client’s own responses, and overall are positive about the technology (Connoly et al., 2020). Backhaus et al. (2012) conducted a systematic review of 65 papers across a range of populations, concluding that videoconferencing was feasible, associated with good user satisfaction and similar clinical outcomes to in‐person therapy delivery. Recent systematic reviews by Berryhill, Culmer, et al. (2019), and Berryhill, Halli‐Tiemey, et al. (2019) have confirmed that there are significant post‐therapy effects on the most commonly used outcome measures of anxiety and depressive symptoms aggregated across different clinical groups.

Findings of overall acceptability and efficacy of videoconferencing have informed the implementation of videoconferencing within services, particularly for programmes delivered to geographically isolated or dispersed individuals (Morriss et al., 2019; Muir et al., 2020; Varker, Brand, Ward, Terhaad, & Phelps, 2018). Now adoption is more widespread, practitioners and clients may need to choose between video and in‐person as more equally available options. To inform clinical decision‐making, it is now important to consider when, and for whom, this mode of therapy delivery may be applicable. This is particularly important when weighing up choices against pandemic‐related health risks associated with transit and in‐person contact, which can be avoided by remote therapy delivery.

This review considers the literature to date on how videoconferencing can be utilized for the delivery of psychological therapy to adults presenting with mental health problems. Extending upon previous reviews, which have considered outcomes and satisfaction with videoconferencing across studies as a whole, we consider the following questions:

- For which mental health populations and psychological interventions is there current evidence for psychological therapy being acceptable and efficacious when delivered via videoconferencing?
- What are client and therapist experiences of psychological therapy delivered by videoconferencing, including perceived benefits, challenges and opportunities?

## Methodology

Primary research studies were identified via the databases PubMed, Medline, PsycINFO, and EMBASE in July 2020. Search terms included combinations of the search terms videoconferencing, telehealth telemedicine, telemental health, telepsychology, telepsychiatry, telepsychotherapy, or telecounselling; psychological therapy, psychotherapy, counselling, psychological intervention, or cognitive behaviour; and mental disorder, mental health, mental illness, anorexia, anxiety, bipolar, bulimia, depression, eating disorder, mood disorder, obsessive–compulsive, personality disorder, post‐traumatic stress, psychosis, and schizophrenia. Database searches were supplemented by review of reference lists of included papers and previous review papers.

Studies were included which focused on adult populations experiencing adult mental disorders or clinically significant symptoms of mental disorder. Studies were excluded that focused on children and/or adolescents; people with mental health problems secondary due to physical illness, substance abuse and/or addictions, neurocognitive disorders, learning difficulties, or intellectual disabilities; healthy populations at risk of developing mental health difficulties; and families of people with mental health difficulties. Studies were included that used one‐to‐one psychological interventions delivered via videoconferencing. This excluded group‐based, couple or family interventions; simulated therapy sessions; self‐help; general psychiatric care; asynchronous psychological therapy delivered via recorded video. Studies were included that reported quantitative or qualitative data relevant to understanding outcomes or experiences of therapy. Case studies were excluded, as were studies that did not disaggregate psychological therapy results from broader findings, but multiple baseline case series were included.

Abstracts were screened by CM, and full papers were independently reviewed against the inclusion criteria by CM and NT. Each author led extraction of data for a component of the review, with all data extraction checked and verified by NT. Data on outcomes and acceptability were extracted and considered within diagnostic groupings, with a main focus on randomized controlled trial findings, and pre‐to‐post studies and case series findings considered when they added to the trial literature. Client and practitioner experiences of videoconferencing were considered across the literature, prioritizing systematically collected data reported by papers, but also including anecdotal participant comments. A thematic synthesis (Lucas, Baird, Arai, Law, & Roberts, 2007) was conducted with a lens of identifying the prominent benefits, challenges, and considerations in delivery.

## Results

The literature search identified 1637 papers once duplicates were removed, with a total of 69 papers reporting on 54 discrete studies meeting inclusion criteria (see Table 1). These included 21 randomized controlled trials (RCTs), 20 pre‐to‐post and non‐randomized comparison trials, 6 case series, 4 stand‐alone qualitative studies, and 3 studies examining rates of uptake. Of the RCTs, 7 examined efficacy compared with a non‐therapy control, and 15 included a head‐to‐head comparison with in‐person therapy, of which 9 conducted formal non‐inferiority or equivalence analyses (detailed in Table 2). No studies contrasted videoconferencing with other remote communication modalities (e.g., telephone).

**Table 1:** Included studies

Study	Population	Country	Design	Comparison	*N*	Therapy	Location	System	Primary outcomes	Acceptability and alliance measures
PTSD										
Acierno et al. (2016), Strachan et al. (2012), Gros et al. (2012)	Veterans with PTSD	USA	RCT	IP (NI)	232	BA‐TE	Home	Own device + provided software, or videophone (Viterion 500)	PCL‐M, BDI	CPOSS, SDPQ
Acierno et al. (2017), Gros, Allan, et al. (2018), Gros, Lancaster, et al. (2018)	Veterans with comorbid PTSD and depression	USA	RCT	IP (NI)	150	PE	Home	Own device + AK Summit software or provided tablet or videophone	CAPS, PCL‐M, BDI	CPOSS, SDPQ
Franklin et al. (2017)	Veterans with PTSD	USA	RCT	TAU	27	PE	Home or clinic	Computer + provided software, or iPhone + Tango	CAPS, PDS	Preferred therapy modality, attrition
Germain et al. (2009), Germain et al. (2010), Marchand et al. (2011)	PTSD	Canada (rural)	NRCT	IP	68	CBT	Clinic	Tandberg 2500 VC units	MPSS	WAI, SEQ, DCCS, VT‐Q, VTS
Gros et al. (2011)	Veterans with PTSD	USA	NRCT	IP	89	PE	Clinic	Tandberg 1000 MXP VC units	PCL‐M	IIRS
Hassija and Gray (2011)	Women with PTSD from domestic violence	USA (rural)	pre–post	‐	15	PE or CPT	Clinic	Polycom VSX3000 VC units	PCL, CES‐D	Satisfaction Questionnaire
Liu et al. (2019)	Veterans with PTSD, male and female	USA	RCT	IP (NI)	207	CPT	Clinic	Not stated	CAPS, PCL‐S, PHQ‐9	‐
Luxton et al. (2015)	Active military and veterans with PTSD	USA	pre–post	‐	10	BA	Home	Laptop + Cisco Jabber	CAPS, PCL‐M, BDI	TSC, CSQ
Maieritsch et al. (2016)	Veterans with PTSD	USA	RCT	IP (E)	90	CPT	Clinic	Not stated	CAPS, PCL	WAI
Morland et al. (2015)	Women with PTSD, civilians and veterans	USA	RCT	IP (NI)	149	CPT	Clinic	Not stated	CAPS	WAI, CPOSS‐VA, TSAS, TEQ
Olden et al. (2017)	PTSD in high‐risk occupations	USA	pre–post	‐	11	PE	Home or clinic	Polycom VC units (clinic) or own device (home)	CAPS, PCL	WAI, CSQ, TSAS, ETO
Tuerk et al. (2010)	Veterans with PTSD	USA (rural)	NRCT	IP	47	PE	Clinic	Tandberg 1000 MXP VC units	PCL‐M, BDI	‐
Yuen et al. (2015)	PTSD, combat related	USA	RCT	IP (NI)	52	PE	Home	Own device/tablet + VC software or videophone	CAPS, PCL‐M	‐
Ziemba et al. (2014)	PTSD	USA	RCT	IP	18	CT	Clinic	Polycom VC units	CAPS	Satisfaction survey
Depression										
Arnaert et al. (2007)	Older adults with depression		qualitative	‐	4	PST	Home	Videophone	‐	Interviews
Jang et al. (2014)	Korean migrants with depression	USA	pre–post	‐	12	CBT	‘Place convenient to client’	Laptop + Vidyo	PHQ‐9	CSQ
Lazzari, Egan, and Rees (2011)	Depression	Australia	pre–post	‐	3	BA	Clinic	Not stated	GDS	Satisfaction questionnaire
Choi, Hegel, et al. (2014), Choi, Marti, et al. (2014), Choi et al. (2013)	Housebound adults over 50 with depression	USA	RCT	IP, TAU	158	PST	Home	Laptop + Skype	HRSD, WHODAS	TEI, interviews
Deen et al. (2013)	Primary care attendees with positive depression screen	USA	uptake study	‐	179	CBT	Home	Not stated	Uptake of therapy	‐
Egede et al. (2015)	Veterans with depression	USA	RCT	IP (NI)	241	BA	Home	Videophone	BDI, GDS, SCID	‐
Luxton et al. (2016), Smolenski et al. (2017), Pruitt et al. (2019)	Military personnel with depression	USA	RCT	IP (NI)	121	BA	Home	Laptop + Cisco Jabber	BDI, BHS	IASMHS
Sayal et al. (2019)	Young adults presenting with self‐harm and depression	UK	RCT	TAU	22	PST	Not stated	Mobile phone or video calling (WebeX)	BDI	Interviews
Lichstein et al. (2013)	Comorbid depression and insomnia	USA	pre–post	‐	5	CBT	Clinic	Laptop + Skype	HRSD, CSD, ISI,	WAI, session ratings, feedback survey
Scogin et al. (2018)	Comorbid depression and insomnia	USA (rural)	RCT	TAU	40	CBT	Clinic	Computer + Skype	HRSD, CSD, ISI, SCID	WAI
Anxiety disorders										
Théberge‐Lapointe et al. (2015)	GAD	Canada	MBCS	‐	5	CBT	Clinic	Tandberg 2500 VC system	PSWQ	‐
Watts et al. (2020)	GAD	Canada	RCT	IP (S)	115	CBT	Clinic	Computer + Tandberg MXP software	Not yet reported	WAI
Bouchard et al. (2000)	Panic disorder/ agoraphobia	Canada	pre–post	‐	8	CBT	Clinic	Tandberg 2000 VC system	P&A	WAI, session ratings
Bouchard et al. (2004)	Panic disorder/ agoraphobia	Canada (remote)	NRCT	IP	21	CBT	Clinic	Tandberg 2500 VC system	Daily diaries, ACG, BSQ, MI	WAI, treatment credibility measure
Morriss et al. (2019)	Health anxiety	UK	RCT	TAU	156	CBT	Clinic	WebeX or telephone	SHAI	‐
Yuen et al. (2013)	Social anxiety	USA	pre–post	‐	24	ABBT	Home	Own device + Skype	SPAI, LSAS, FNE	WAI, RTQ, therapist survey
Obsessive–Compulsive										
Fitt and Rees (2012)	OCD	Australia	MBCS	‐	4	MCT	Clinic	Computer + Polycom PVX v 8.0.2	Y‐BOCS	WAI
Himle et al. (2006)	OCD	USA	MBCS	‐	3	CBT	Not stated	Polycom Viewstation VC units	Y‐BOCS	WAI, VTS, satisfaction questionnaire
Goetter et al. (2014)	OCD	USA	pre–post	‐	15	ERP	Home	Not stated	Y‐BOCS	RTQ, WAI, CSS, VTS, PEAS
Vogel et al. (2014)	OCD	Norway	RCT	SH, TAU	30	ERP	Clinic	Computer/tablet + FaceTime	ADIS‐IV, Y‐BOCS, VOCI	WAI, VTS
Muroff and Steketee (2018)	Hoarding	USA	case series	‐	7	CBT	Home	Own device + VC software	HRS‐I, SI‐R	WAI
Lee et al. (2018)	Trichotillomania	USA	RCT	TAU	22	ACT + HRT	Home	VSee software (device not stated)	MGH‐HPS	WAI, CSQ
Eating disorders										
Abrahamsson et al. (2018)	Binge eating disorder and obesity	Sweden (rural)	case series	‐	5	CBT	Not stated	Mobile device + VC software	Meal frequency (EDE)	WAI, CSQ, SUS
Giel et al. (2015)	Anorexia nervosa	Germany	pre–post	‐	16	MM	Not stated	Laptop + Cisco VC software	BMI, SCID diagnosis, EDE	Satisfaction ratings
Hamatani et al. (2019)	Bulimia nervosa or binge eating disorder	Japan	pre–post	‐	7	CBT	Home	Cisco WebEx (device not stated)	EDE	WAI
Mitchell et al. (2008); Ertelt et al. (2011); Marrone et al. (2009)	Bulimia nervosa or EDNOS	USA	RCT	IP	128	CBT	Clinic	Telemedicine equipment (model not specified)	EDE	WAI, HPRS
Simpson et al. (2005, 2006)	Bulimia nervosa or EDNOS	UK (remote)	case series	‐	6	CBT	Clinic	Sony 1600 VC units + VC software	SEDS, BEI‐II, BITE	ARM, satisfaction survey, interview
Yu et al. (2020)	Binge eating disorder	USA	RCT	IP	18	CBT	Not stated	Own device + Fruit Street	EQE, EAT, TFEQ, YFAS	Satisfaction ratings
Mixed diagnoses										
Brunnbauer et al. (2016)	Psychology clinic referrals, mixed diagnoses	Australia	pre–post	‐	20	Individualized	Not stated	Not stated	CORE‐10, DASS	‐
Dunstan and Tooth (2012)	Mood or anxiety disorder	Australia	pre–post	‐	8	Individualized	Clinic	Video monitor + VC software	SUDS, DASS, OQ45	Interviews
Griffiths et al. (2006)	Mood or anxiety disorder	Australia (rural)	pre–post	‐	15	CBT	Clinic	Computer + VC software	MHI, HoNOS	Satisfaction rating
Gonzalez and Brossart (2015)	Rural residents, mixed diagnoses	USA (rural)	pre–post	‐	52	Individualized	Clinic	Not stated	CORE, PHQ‐9, SF‐12	‐
Lindsay et al. (2015)	Veterans, mixed diagnoses	USA (rural)	qualitative	‐	93	Individualized	Home	Not stated	Practitioner and client interviews	‐
Matsumoto et al. (2018, 2020)	Mixed diagnoses	Japan	pre–post	‐	30	CBT	Home	iPad Mini + Cisco WebEx	Y‐BOCS, PDSS, LSAS	WAI
Morgan et al. (2008)	People in prison or secure psychiatric hospital, mixed diagnoses	USA	RCT	IP	186	Individualized	Prison/ Hospital	Not stated	‐	WAI, SEQ, CSQ
Simpson et al. (2001), Simpson (2001)	People living in a remote area, mixed diagnoses	UK (remote)	qualitative	‐	10	Individualized	Clinic	Computer + VC software	Interview	PHAS
Simpson et al. (2015)	Psychology clinic referrals, mixed diagnoses	Australia	qualitative	‐	6	CBT	Clinic	Computer + Cisco C20 endpoint	Interview, CORE	ARM
Stubbings et al. (2013)	Mood or anxiety disorder	Australia	RCT	IP	26	CBT	Clinic	Computer + iChat	DASS, QLESQ	WAI, CSQ, TSQ
Valentine, Donofry, and Sexton (2020)	Veterans, mixed diagnoses	USA	uptake/ retention	‐	250	CBT	Clinic/ home	Not stated	Uptake of VC	‐
Yang et al. (2019)	Psychotherapy referrals with post‐partum mood or anxiety disorder	Canada	uptake (RCT design)	IP	38	CBT	Home	Own device + VC software	Uptake of VC, EPDS	TSQ, patient reported costs

Note

VC videoconferencing. Populations: EDNOS = eating disorder not otherwise specified; GAD = generalized anxiety disorder; OCD = obsessive–compulsive disorder; PTSD = post‐traumatic stress disorder. Design: MBCS = multiple baseline case series; NRCT = non‐randomized controlled trial; RCT = randomized controlled trial. Comparison: IP = in‐person; IP (E) = in‐person, including an equivalence analysis; IP (NI) = in‐person, including a non‐inferiority analysis. TAU = treatment‐as‐usual or enhanced treatment‐as‐usual condition; SH = self‐help. Therapies: ABBT = acceptance‐based behaviour therapy; BA = behavioural activation; BA‐TE = behavioural activation and therapeutic exposure; CPT = cognitive processing therapy; CT = cognitive therapy; HRT = habit reversal therapy; MM = Maudsley model; PE = prolonged exposure; ERP = exposure and response prevention; MCT = metacognitive therapy; PST = problem‐solving therapy. Measures: ACQ = Agoraphobic Cognitions Questionnaire; ADIS‐IV = Anxiety Disorders Interview for DSM‐IV; BDI Beck = Depression Inventory; BHS = Beck Hopelessness Scale; BITE = Bulimic Investigatory Test; BMI = body mass index; BSQ = Body Sensation Questionnaire; CAPS = Clinical Administered PTSD Scale; CES‐D = Centre for Epidemiology Scale for Depression; CORE = Clinical Outcomes Routine Evaluation; CPOSS = Charleston Psychiatric Outpatient Satisfaction Scale; CSD = Consensus Sleep Diary; CSQ = Client Satisfaction Questionnaire; CSS = Client Satisfaction Survey; DASS = Depression Anxiety Stress Scale; DCCS = Distance Communication Comfort Scale; EAT = Eating Attitude Test; EDE = Eating Disorder Examination; EPDS = Edinburgh Postnatal Depression Scale; ETO = Expectancy of Therapeutic Outcome; GDS = Geriatric Depression Scale; HPRS = Hill Process Rating System; HRSD = Hamilton Rating Scale for Depression; HRS‐I = Hoarding Rating Scale‐Interview; IASMHS = Inventory of Attitudes Toward Seeking Mental Health Service; ISI = Insomnia Severity Index; LSAS = Liebowitz Social Anxiety Scale; MGH‐HPS = Massachusetts General Hospital Hair Pulling Scale; MHI = Mental health Inventory; MI = Mobility Inventory for Agoraphobia; MPSS = Modified PTSD Symptom Scale; OQ45 = Outcome Questionnaire 45; P&A = Panic and Agoraphobia Scale, PCL PTSD Checklist (M Military version) ; PDS = Posttraumatic Diagnostic Scale; PDSS = Panic Disorder Severity Scale; PEAS = Patient EX/RP Adherence Scale; PHAS = Penn Helping Alliance Scale; PHQ‐9 = Patient Health Questionnaire for Depression; PSWQ = Penn State Worry Questionnaire; QLESQ = Quality of Life Enjoyment and Satisfaction Questionnaire‐18 item; RTQ = Reaction to Treatment Questionnaire; SCID = Structured Clinical Interview for DSM; SDPQ = Service Delivery Perception Questionnaire; SEDS = Survey for Eating Disorders; SEQ = Session Evaluation Questionnaire; SHAI = Short Health Anxiety Inventory; SI‐R = Saving Inventory Revised; SPAI = Social Phobia and Anxiety Inventory; SUDS = Subjective Units of Distress Scale; TEI = Treatment Evaluation Inventory; TEQ = Treatment Expectancy Questionnaire; TFEQ = Three‐Factor Eating Questionnaire; TSAS = Telemedicine Satisfaction and Acceptance Scale; TSC = Treatment Session Checklist; TSQ = Telehealth Satisfaction Questionnaire; VOCI = Vancouver Obsessional Compulsive Inventory; VTF = Videoconference Therapy Questionnaire; VTS = Videoconferencing Telepresence Scale; WAI = Working Alliance Inventory; WHODAS = World Health Organization Disability Scale; YAFS = Yale Food Addiction Scale; Y‐BOCS = Yale‐Brown Obsessive–Compulsive Scale.

**Table 2:** Between group differences in working alliance, primary outcomes, dropout, and satisfaction in large randomized controlled trials involving direct comparisons with in‐person therapy

Study	Population	*N*	Therapy	Therapeutic alliance	Primary outcome	Dropout/satisfaction
Acierno et al. (2016)	PTSD and depression, veterans	232	BA‐TE	‐	PCL‐M, BDI: VC non‐inferior to IP at post‐therapy, and 3 and 12 months	Rate of completion of both therapy and post‐treatment assessment: no difference (VC 82%, IP 77%)
Acierno et al. (2017), Gros, Allan, et al. (2018)	PTSD and depression, veterans	150	PE	‐	PCL‐M: VC non‐inferior to IP at post‐therapy, 3 months and 6 months; BDI: VC non‐inferior to IP at 6 months, inconclusive at post and 3 months	No difference in number of sessions attended (VC 7.6, IP 8.6) or completion of a minimum dose of 6 sessions, but discontinuation occurred earlier in VC over sessions 1‐8
Liu et al. (2019)	PTSD, veterans	207	CPT	‐	CAPS: VC non‐inferior to IP at 6 months, but not at post‐therapy; PCL: VC non‐inferior to IP at post and 6 months. PHQ‐9: VC non‐inferior to IP at post and 6 months	No difference in study dropout (VC 23%, IP 28%)
Maieritsch et al. (2016)	PTSD, veterans	90	CPT	WAI client ratings show equivalence	CAPS, PCL: inconclusive but trend for equivalence between groups (*p* < .10)	High rates of treatment dropout (43% overall) but no difference by group
Morland et al. (2015)	PTSD, female, civilians and veterans	149	CPT	WAI client ratings: VC inferior to IP at session 2, but difference small (*d* = −0.07), and no difference at session 6 or 12; therapist ratings: no difference at any time point. Homework completion: no difference (VC 77%, IP 80%)	CAPS: VC non‐inferior to IP at post‐treatment, 3 and 6 months.	Therapy completion rate: **no difference** (≥ 10 sessions: VC 76%, IP 79%). Treatment expectations: no difference. Satisfaction ratings: both groups rated service highly on global ratings, with no difference, but VC inferior to IP on CPOSS ratings of broader service delivery (*d* = −0.24)
Yuen et al. (2015)	PTSD, combat‐related	52	PE	No difference on ratings of how comfortable feel talking with therapist or quality of communication	CAPS: VC non‐inferior to IP; PCL: neither group superior but non‐inferiority analysis inconclusive	SDPQ: 100% satisfied with treatment in both VC and IP
Choi, Hegel, et al. (2014), Choi, Marti, et al. (2014)	Depression, housebound adults over 50	158	PST	‐	HAMD: neither group superior at 12 or 24 weeks; VC superior to IP at 36 weeks; WHODAS: neither superior at any time point	Treatment Evaluation Inventory: VC superior to IP
Egede et al. (2015)	Depression, veterans	241	BA	‐	BDI, GDS, SCID: VC non‐inferior at 4 weeks (mid), 8 weeks (post) and 3 months.	No difference in full therapy completion rate (VC 81%, IP 79%)
Luxton et al. (2016)	Depression, military personnel	121	BA	‐	BDI: VC non‐inferior to IP at mid‐therapy and 12 weeks, but not at post‐therapy; BHS: VC non‐inferiority not established at any time point, and found to be inferior to IP at post‐therapy.	CSQ: high satisfaction, no difference between groups. Attitudes to seeking mental health treatment: no difference between groups. No difference in full therapy completion rate (VC 64%, IP 71%)
Watts et al. (2020)	Generalized anxiety disorder	115	CBT	WAI: Across 8 time points, VC superior to IP in client ratings; neither group superior on therapists’ ratings.	‐	‐
Mitchell et al. (2008), Ertelt et al. (2011), Marrone et al. (2009)	Bulimia nervosa or EDNOS	128	CBT	WAI: no difference in client ratings, VC inferior to IP in therapist ratings.	EDE: neither group superior for abstinence from bingeing and/or purging. VC inferior to IP for reduction in binge eating frequency across time points.	Client ratings of treatment suitability, client expectation of success, and number of sessions completed: no difference.
Morgan et al. (2008)	People in prison or secure forensic psychiatric hospital	186	Individualized therapy	WAI: no difference in client ratings.	‐	No differences on CSQ, or ratings of session depth, smoothness, positivity or distress.

Note:

Only includes randomized controlled trials sufficiently powered to detect large between group effects (*N ≥ *52 at 80% power). If no primary outcome specified, symptoms of target disorder listed. *Superiority/inferiority* refers to group differences observed versus a null hypothesis of *no difference*; *non‐inferiority* (one tailed test) and *equivalence* (two‐tailed test) refer to whether or not the confidence interval for the difference includes a null hypothesis of the groups differing by the minimum clinically significant difference. PTSD = post‐traumatic stress disorder; EDNOS = Eating disorder not otherwise specified. Therapies: BA = behavioural activation; BA‐TE = behavioural activation and therapeutic exposure; CBT = cognitive behavioural therapy; CPT = cognitive processing therapy; PST = problem‐solving therapy. Groups: IP = in‐person; VC = videoconferencing. Measures: BDI = Beck Depression Inventory; CAPS = Clinician Administered PTSD Scale; CGI = Clinical Global Impression; CPOSS = Charleston Psychiatric Outpatient Satisfaction Scale; CSQ = Client Satisfaction Questionnaire; EDE = Eating Disorders Examination; GDS = Geriatric Depression Scale; HAMD = Hamilton Rating Scale for Depression; SCID = Structured Clinical Interview for DSM diagnosis; SCL‐90R = Hopkins Symptom Checklist; SDPQ = Service Delivery Perceptions Questionnaire; SEQ = Session Evaluation Questionnaire; WAI = Working Alliance Inventory.

The most frequently studied diagnostic groups were post‐traumatic stress disorder (PTSD; 14 studies), and depression (10), for which there were a number of well‐powered RCTs, followed by anxiety disorders (6), obsessive–compulsive spectrum disorders (6) and eating disorders (6). Twelve additional studies examined mixed diagnosis populations including a large RCT. Across these studies, a number examined implementations to specific populations, with a large number, particularly PTSD studies, conducted with veterans or military personnel, and others focusing on populations with difficulties attending clinic settings in person, including people with difficulties leaving the home, people living in rural or remote areas, prison inmates, and geographically dispersed members of migrant populations. Less than half of studies were conducted within the person’s home/residence, with many especially older studies, involving visiting a local clinic using telehealth equipment to connect with a therapist in a different location. The types of technology used for videoconferencing included dedicated telemedicine hardware, analogue videophones, and, increasingly, using Internet‐based videoconferencing software on computers or smartphones. Many studies provided participants with equipment such as laptop or tablet computers, but more recent studies have used participants’ own devices.

Across the full range of studies, therapy was found feasible to deliver via videoconferencing, clients were satisfied with therapy, and expected improvements in targeted symptoms occurred. We consider the findings for specific populations in detail (summarized in Table 3), followed by broader findings about use of videoconferencing across all studies.

**Table 3:** Summary of evidence for feasibility, acceptability, and efficacy by population

	Therapy models found feasible to deliver using videoconferencing	Outcomes of videoconferencing delivery
PTSD	BA‐based exposure therapy, CPT, prolonged exposure	6 of 7 RCTs found non‐inferior to in‐person therapy, with the other finding videoconferencing inferior at post‐treatment and non‐inferior at follow‐up
Depression	BA, CBT, problem‐solving therapy	1 RCT found superior to routine care. 3 RCTs compared with in‐person therapy, finding few differences between modalities, and 1 trial establishing non‐inferiority.
Anxiety disorders	CBT, including focused therapies for GAD, panic disorder, social anxiety and health anxiety	1 RCT, with health anxiety, found superior to routine care. Pre–post studies show improvements following therapy for other anxiety disorders. No non‐inferiority trials conducted, but 1 small RCT (mixed diagnoses) found similar outcomes to in‐person delivery.
Obsessive–compulsive disorders	CBT, ERP; CBT for hoarding; habit reversal therapy for trichotillomania	No fully powered RCTs. Pre–post studies show improvements following therapy.
Eating disorders	CBT; Maudsley Model‐based relapse prevention	1 RCT (bulimia nervosa) comparing to in‐person therapy, finding few differences. Pre–post improvements observed for both bulimia nervosa and anorexia nervosa
Psychotic disorders	No studies identified	‐
Bipolar disorder	No studies identified	‐
Personality disorders	No studies identified	‐

Note:

BA = behavioural activation; CBT = cognitive behaviour therapy; CPT = cognitive processing therapy; ERP = exposure and response prevention; RCT = randomized controlled trial.

### Application of videoconferencing with different populations

#### Post‐traumatic stress disorder

PTSD was the most researched mental health diagnosis. In addition to small pre–post studies and pilot RCTs, the search identified 7 well‐powered RCTs of videoconferencing therapy for PTSD, covering a range of treatment protocols, including cognitive processing therapy (CPT), prolonged exposure (PE), and behavioural activation.

Two trials examined the use of the eight‐to‐twelve session PE protocol to treat PTSD in veterans (Acierno et al., 2017, also reported on in Gros, Allan, Lancaster, Szafranski, & Acierno, 2018; Gros, Lancaster, López, & Acierno, 2018; and Yuen et al., 2015), and one trial combined behavioural activation with exposure therapy to treat both PTSD and depression (Acierno et al., 2016; Gros et al., 2012; Strachan, Gros, Ruggiero, Lejuez, & Acierno, 2012). All compared videoconferencing to in‐person delivery and had samples that were over 90% male. Videoconferencing showed similar rates of therapy completion (Acierno et al., 2016, 2017; Yuen et al., 2015) and satisfaction (Gros, Allan, et al., 2018; Yuen et al., 2015) and was non‐inferior to in‐person for PTSD, depression, and anxiety (Acierno et al., 2016, 2017; Yuen et al., 2015).

Four trials examined CPT delivered by videoconferencing in comparison with in‐person therapy (Glassman et al., 2019; Lui et al., 2019; Maieritsch et al., 2016; Morland et al., 2015). Participants were again predominantly veterans, with one study also including civilians (Morland et al., 2015), but females were better represented in CPT studies (Lui et al., 2019: 45% female, Morland et al., 2015: 100% female). Delivery by videoconferencing was found to be non‐inferior to in‐person in reducing PTSD symptoms in all studies other than Lui et al. (2019) who found that videoconferencing was inferior at post‐treatment, but equivalent at 6‐month follow‐up. All studies found no significant differences in dropout or satisfaction between videoconferencing and in‐person conditions.

Overall, the generally positive findings of acceptability and efficacy of videoconferencing for exposure‐based therapies are noteworthy, suggesting this modality is able to support this emotionally challenging, experientially focused, treatment. It has also been observed that videoconferencing clients rate the therapeutic alliance as highly for exposure‐based sessions as other CBT‐based sessions (Germain, Marchand, Bouchard, Guay, & Drouin, 2010).

#### Depression

We identified 3 well‐powered RCTs of videoconferencing therapy for depression, 3 smaller RCTs, and 4 studies using other designs. Two studies, including one of the RCTs (Yang, Vigod, & Hensel, 2019), primarily reported on uptake of videoconferencing. Intervention models included problem‐solving therapy, behavioural activation and combined CBT protocols for depression with insomnia, and for depression with self‐harm. Overall, results suggested participants were satisfied with therapy, and ratings of acceptability and efficacy appeared similar to in‐person delivery.

Problem‐solving therapy was examined in a three‐arm RCT which compared videoconferencing or in‐person delivery with a supportive weekly care‐call control condition in 158 housebound adults over the age of 50 with depression (Choi, Hegel, et al., 2014; Choi, Marti, et al., 2014). On the Hamilton Rating Scale for Depression (HRSD), both videoconferencing and in‐person problem‐solving therapy were superior to the control condition at 12 weeks, 24 weeks, without differing from each other, and videoconferencing was superior to both conditions at 36 weeks (Choi, Marti, et al., 2014).

Videoconferencing‐based behavioural activation has been examined in two RCTs, both conducted with veterans. Luxton et al. (2016) conducted an RCT of an 8‐session behavioural activation intervention delivered by telehealth or in‐person to 121 military personnel and veterans with depression. Both conditions showed significant post‐treatment improvements on the Beck Depression Inventory (BDI) as the primary outcome, and non‐inferiority analyses showed videoconferencing was non‐inferior at mid‐treatment and 12‐week follow‐up, but not immediately post‐therapy. Egede et al. (2015) obtained more conclusive results in a larger non‐inferiority trial with 241 older veterans with major depression. Comparing videoconferencing delivery using a videophone system with in‐person delivery, non‐inferiority was established with no significant differences observed in trajectories of improvement on the BDI and Geriatric Depression Scale, with rates of recovery similar between conditions.

Smaller studies have additionally demonstrated feasibility and acceptability of delivering CBT‐based therapies via videoconferencing to specific populations such as women with post‐partum depression or anxiety (Yang et al., 2019) and Korean migrants with depression (Jang et al., 2014). Among other notable studies, Scogin et al. (2018) conducted a small RCT of a 10‐session CBT‐based treatment for comorbid depression and insomnia delivered via Skype, which found superiority over usual care on a measure of insomnia, but not the HRSD. Finally, in treating self‐harm, Sayal et al. (2019) commenced a small RCT (*N* = 22) of problem‐solving therapy for young adults following presentation for self‐harm and mild depression. However, this was discontinued due to recruitment difficulties (an analysis of which did not attribute these to the use of videoconferencing).

#### Anxiety disorders

Anxiety disorders have been less fully studied than depression. Nonetheless, anxiety disorders feature as a major group in a number of mixed diagnosis studies, which have demonstrated that CBT‐based therapies can be satisfactorily delivered (e.g., Brunnbauer et al., 2016; Dunstan & Tooth, 2012; Griffiths, Blignault, & Yellowlees, 2006; Matsumoto et al., 2018, 2020; Stubbings et al., 2013). Among these, an RCT design was used by Stubbings, Rees, Roberts, and Kane (2013) in a study of 26 people with mainly anxiety disorders. Reductions on all subscales of the Depression Anxiety Stress Scale (DASS) were observed following videoconferencing CBT, and, while underpowered, no differences in the magnitude of effect were observed between videoconferencing and an in‐person comparison group. The feasibility of applying videoconferencing to deliver therapies to other specific populations is indicated by the following, mainly small, studies.

#### Generalized anxiety disorder (GAD)

A multiple baseline case series by Théberge‐Lapointe, Marchand, Langlois, Gosselin, and Watts (2015) showed evidence for successful cognitive behavioural treatment of GAD, with five participants no longer meeting diagnostic criteria post‐therapy and 3 months later, and this outcome persisting to 12 months after treatment in all but one case. At the time of writing, initial results from a large RCT of CBT for GAD (*N* = 115), focusing on working alliance, have been reported by Watts et al. (2020), with clients rating the working alliance more highly for videoconferencing than in‐person therapy across time points, although therapists rated both modes of delivery similarly.

*Panic disorder and agoraphobia* have only been studied in small pre‐to‐post studies, all of CBT. Bouchard et al. (2000) found significant improvements across all measures, reporting that five out of the eight participants no longer experienced panic attacks after the 12‐week treatment. Bouchard et al. (2004) delivered the same intervention to a further 10 videoconferencing cases, compared with a non‐randomized in‐person delivery group. Nearly all participants achieved remission at the end of treatment, maintained six months later, a similar to in‐person delivery. Matsumoto et al. (2018) also found significant reductions in panic symptoms among 10 participants with panic disorder in their pre‐to‐post study of CBT.

##### Social anxiety

Modality of delivery is of particular interest for social anxiety, where communication itself is a source of anxiety. Yuen et al. (2013) examined 12 sessions of acceptance‐based behaviour therapy for 24 individuals with SAD. Therapists rated the use of videoconferencing as feasible, and there were post‐therapy improvements on several questionnaire measures of social anxiety, maintained and at the 3‐month follow‐up, as well as changes on observer‐rated social behaviour; participants indicated that they were satisfied with the treatment. Likewise, Matsumoto et al. (2018) found reductions in social anxiety following videoconferencing‐based CBT in their small sample of 10 social anxiety participants.

##### Health anxiety

The largest study for a specific anxiety disorder has been for health anxiety: Morris et al. (2019) conducted an RCT comparing CBT delivered via videoconferencing or telephone with routine care in 156 participants. Supporting the use of videoconferencing, health anxiety was reduced in the therapy group relative to routine care at 6‐, 9‐, and 12‐month time points.

#### Obsessive–compulsive and related disorders

Research into videoconferencing‐delivered psychological treatments in obsessive–compulsive and related disorders was limited, with studies limited to case series and small sample single‐arm open trials and pilot RCTs. Nevertheless, there is an emerging support for the acceptability and effectiveness of videoconferencing for a range of intervention types across OCD, hoarding and trichotillomania.

Matsumoto et al. (2018) reported on a standard 16‐week CBT treatment for their 10 OCD patients. Symptom reduction pre–post treatment, strong therapeutic alliance, high rates of satisfaction with treatment, and 100% retention, supported the effectiveness and feasibility of the intervention. Further, two studies (Goetter, Herbert, Forman, Yuen, & Thomas, 2014; Vogel et al., 2014) successfully used exposure and response prevention (ERP) to treat OCD via videoconferencing, with post‐treatment symptom reductions. Vogel et al. (2014) noted high engagement with treatment, an ability to observe exposure exercises as they occur in participants’ natural environments, and an opportunity to involve family members and carers, thus addressing family accommodation to rituals where appropriate.

Emerging investigations in hoarding and trichotillomania provide support for its effectiveness, feasibility, and that it provides additional benefits when compared to existing treatments. Muroff and Steketee (2018) delivered a structured CBT treatment for seven patients with hoarding. Six of the seven patients experienced improvements in symptoms post‐treatment, with five maintaining the gains at 3‐month follow‐up. The ability to use portable devices to move around rooms was noted as an important facilitator in the treatment. In relation to trichotillomania, Lee, Haeger, Levin, Ong, and Twohig (2018) conducted an RCT comparing videoconferencing‐based ACT‐enhanced Habit Reversal Therapy to waitlist control in 22 trichotillomania patients. The study had high retention rates with only one dropout in each condition, and high levels of participant satisfaction and therapeutic alliance. Statistically and clinically significant improvements in trichotillomania symptoms were noted among the treatment group.

#### Eating disorders

In the treatment of eating disorders, there has been a single large RCT, which examined CBT for bulimia nervosa and related disorders (Ertelt et al., 2011; Marrone, Mitchell, Crosby, Wonderlich, & Jollie‐Trottier, 2009; Mitchell et al., 2008). Although bulimia symptoms reduced for both videoconferencing and in‐person delivery, and rates of abstinence from bingeing and/or purging showed were similar, the reduction in binge eating frequency was less for videoconferencing participants across multiple time points (Mitchell et al., 2008). Working alliance was rated similarly by clients for each of the conditions, but therapists rated the alliance less strongly in the videoconferencing condition (Ertelt et al., 2011).

Most other studies identified by the search examined smaller single group samples for bulimia and related disorders, reporting reductions in bulimic symptoms (Abrahamssom, Ahlund, Ahrin, & Alfonsson, 2018; Hamatani et al., 2019; Simpson et al., 2006) and satisfaction with the online modality (Abrahamssom et al., 2018; Simpson et al., 2005).

For anorexia nervosa, Giel et al. (2015) conducted a single group pilot study examining a relapse prevention intervention based on the Maudsley model (Schmidt, Magill, & Renwick, 2015) in 16 individuals. Eight sessions were delivered via videoconferencing, bookended by two in‐person sessions. Three‐quarters of participants completed therapy, rating high satisfaction, and at post‐intervention body mass index had increased by an average of 1.1 points, eating concerns were reduced, and two participants were in complete remission.

#### Other populations

No studies were identified providing data on videoconferencing therapy delivery to persons with psychotic disorders, bipolar disorder, or personality disorder.

### Client and practitioner experience

#### Overall acceptability

Every RCT comparing at‐home videoconferencing with in‐person delivery at a clinic reported no group differences on questionnaire measures of satisfaction (see Table 2). Differences in satisfaction or dropout were only seen in two studies overall, both delivering interventions within the same environment: Morland et al. (2015) reported lower satisfaction ratings primarily related to negative experiences of the clinic setting that was attended for videoconferencing (also used in the in‐person condition), suggesting specificity to the potentially impersonal experience of attending a clinic for a video‐based appointment. Conversely, Choi, Hegel, et al. (2014) found that housebound people with depression receiving in‐home therapy via videoconferencing were more satisfied than those being visited by a therapist. Overall, this demonstrates that satisfaction with videoconferencing‐based therapy is as high as traditional forms of delivery.

In terms of therapy dropout, nearly all comparisons with in‐person therapy revealed no group differences (see Table 2). An exception was a follow‐up analysis of discontinuation in the trial by Acierno et al. (2017) reported that early dropout tended to arise more often with videoconferencing (Gros, Allan, et al., 2018), even though overall session attendance rates were similar. While dropout seems to only arise in a small number of people, other studies report discomfort with videoconferencing being cited by participants as a reason for dropout, so this may be an issue with a small number of people, although at this stage there is a lack of information on what contributes to this (Germain, Marchand, Bouchard, Drouin, & Guay, 2009; Lichstein et al., 2013; Simpson, Bell, Knox, Mitchell, & Eating, 2005).

It should be noted that individual comments expressing a *preference for in‐person therapy* were often noted from videoconferencing participants (Choi, Wilson, Sirrianni, Marinucci, & Hegel, 2013; Lichstein et al., 2013; Simpson et al. 2005, 2006). Among qualitative client reports, a period of *early discomfort and adaptation* to using videoconferencing technology was also an experience reported by participants across studies (Choi, Hegel, et al., 2014; Dunstan & Tooth, 2012; Fitt & Rees, 2012; Germain et al., 2009; Lichstein et al., 2013; Simpson et al., 2005, 2006; Simpson et al., 2015; Yuen et al., 2015). For some participants, attitudes towards videoconferencing (including scepticism, anxiety, unfamiliarity) were linked to the experiences of discomfort in early sessions (Arnaet, Klooster, & Chow, 2007; Choi, Hegel, et al., 2014; Fitt & Rees, 2012; Simpson et al., 2005, 2006). For most, this *early discomfort was reduced over time*, as participants got more comfortable with the technology (Choi, Hegel, et al., 2014; Dunstan & Tooth, 2012; Simpson et al., 2005, 2006; Simpson et al., 2015) or their interactions with their therapist became more ‘natural’ (Yuen et al., 2015), although this did not always occur (Choi, Hegel, et al., 2014; Choi et al., 2013; Lichstein et al., 2013; Simpson et al., 2005). Therapists reported similar experiences of initial apprehension and discomfort, before becoming more confident in using videoconferencing technology and adapting to the modality (Dunstan & Tooth, 2012; Michell et al., 2008). This is balanced by other reports of participants *embracing the novelty and use of technology* in therapy delivery (e.g., Aranaet et al., 2007; Choi, Hegel, et al., 2014; Choi et al., 2013; Dunstan & Tooth, 2012). It should be noted that many of these studies were conducted before the widespread day‐to‐day use of videoconferencing platforms, and less adaptation may be required in the 2020s.

#### Facilitating access

One of the presumed benefits of videoconferencing is that it facilitates access. As shown in Table 1, many of the studies reviewed targeted participants in *rural or geographically remote areas*, and some involved applications to potentially *isolated groups* (e.g., housebound older adults; victims of domestic violence, migrants). Participant reports indicated that many people receiving videoconferencing therapy would otherwise have been unable to access any therapy (Choi et al., 2013; Hassija & Gray, 2011), while others included references to challenges of *travel distance* and its associated *financial impact* (Abrahamsson et al., 2018; Simpson et al., 2005; Simpson et al. 2015). Some studies also referred to the opportunity to provide *specialist services* for a specific issue to people over a broad area (Hassija & Gray, 2011; Lee et al., 2018).

Even when not an absolute barrier, the increased accessibility appeared valued. The post‐partum mental health study by Yang et al. (2019) examined uptake when the option to use videoconferencing in place of in‐person psychotherapy sessions was offered: 74% used videoconferencing for at least one therapy session, with 21% doing all therapy via video; *Time and cost savings* were identified, and participants reported being *able to attend* more frequently. In other studies, participants spoke of *convenience*, such as fitting therapy into busy life schedules (Abrahamsson et al., 2018; Choi, Hegel, et al., 2014; Choi et al., 2013; Lee et al., 2018; Yuen et al., 2015), and being able to access therapy from home (Choi, Hegel, et al., 2014). *Continuity of care* independent of location was also highlighted, both in relation to moving house (Simpson et al., 2005, 2006), and being released from prison (Morgan, Patrick, & Magaletta, 2008).

Symptoms of *anxiety*, concerns about *stigma*, and negative thought processes also featured as potential barriers to accessing in‐person services that videoconferencing was able to circumvent (Abrahamsson et al., 2018; Bouchard et al., 2000; Simpson, Guerrini, & Rochford, 2015). For example, in the trichotillomania study by Lee et al. (2018), 40% of participants reported that they would not have entered treatment in an in‐person setting due to shame. *Privacy* for persons in small or rural communities was also referred to (Simpson et al., 2005; Simpson et al., 2015). Nonetheless, privacy was not always assured by videoconferencing with concerns about *privacy from others within the person’s own home* being raised by some participants (Abrahamsson et al., 2018; Choi, Hegel, et al., 2014; Franklin, Cuccurullo, Walton, Arseneau, & Petersen, 2017). Notably, concerns about privacy from use of networked digital technology did not tend to be reported.

#### Client factors predicting uptake and satisfaction

Studies of client variables predicting uptake, engagement and completion of therapy have identified relatively few predictors. In considering predictors of uptake among American primary care attendees with a positive depression screen, Deen, Fortney, and Schroeder (2013) found that uptake of videoconferencing‐based CBT was predicted by perceiving illness to be persisting, believing that treatment would be effective, and reporting *geographic barriers* to attending; Time barriers, financial barriers, perceived stigma, and other beliefs about depression were unrelated to uptake. In a mixed diagnosis veteran sample offered therapy, Valentine et al. (2020) found that videoconferencing therapy uptake, and sessions completed, were each unrelated to age, race, gender, and marital status.

Several studies have examined predictors of differential satisfaction with, or dropout from, videoconferencing therapy. In most studies, completion of therapy appears unrelated to baseline demographic (age, gender, ethnicity, income) and clinical variables (Choi, Hegel, et al., 2014; Germain et al. 2009; Luxton et al., 2016; Watts et al., 2020), although unreplicated findings reported by single studies include greater completion rates for mood rather than anxiety disorders (Valentine et al., 2018), lower baseline PTSD and absence of disability status (Gros, Allan, et al., 2018), and, among veteran samples, being an older, Vietnam‐era veteran (Gros, Yoder, Tuerk, Lozano, & Acierno, 2011). Pruitt et al. (2019) also found that satisfaction with therapy was higher for older military, although, in their sample, this was confounded with serving vs veteran status, with active military needing to travel off base to access videoconferencing facilities. Analyses of predictors of outcome have been limited, but in a military sample, Smolenski, Pruitt, Vuletic, Luxton, and Gahm (2017) found greater baseline anxiety and loneliness predicted participants having a better outcome from in‐person than from videoconferencing‐based therapy.

Among their participants with depression, Choi, Hegel, et al. (2014) found no relationship between ratings of treatment acceptability and computer/Internet ownership, or network quality. Similarly, in an analysis of PTSD trial data, Price and Gros (2014) observed that outcome of PTSD treatment via telehealth was unrelated to prior experience with, or expressed comfort with, telehealth at the outset of treatment. This suggests that prior experience is not a requirement to benefit. However, *prior experience of therapy* appears to predict completion. In their study of uptake, Deen et al. (2013) found predictors of treatment completion were different from those for uptake, and completion was most related to engagement with other treatments: receipt of prior counselling and being prescribed antidepressant medication. Watts et al. (2020) also found prior therapy experience predicted completion.

#### Technical issues

Most studies referred to technical issues as an experience impacting on the delivery of therapy. These included difficulties establishing connection, disconnection, suboptimal audio and visual quality, and bandwidth and connection stability issues resulting in lag and frozen images. Participants considered minor disruptions such as lag as a frustrating and distracting disadvantage of videoconferencing, but, overall, this did not negatively impact on participant engagement (Abrahamsson et al., 2018; Choi et al., 2013; Dunstan & Tooth, 2012; Lichstein et al., 2013). Studies resolved these issues through in‐session troubleshooting or reconnection. Severe technical issues (e.g., disconnection and inability to re‐establish connection) were managed by postponing or cancelling scheduled sessions, or by utilizing a back‐up communication method (e.g., telephone) (Abrahamsson et al., 2018; Germain et al., 2010; Hassija and Gray, 2011; Lee et al., 2018; Luxton, Pruitt, O'Brien, & Kramer, 2015; Olden et al., 2017; Yu et al., 2020; Vogel et al., 2014; Watts et al., 2020).

To proactively manage technical issues, test calls or in‐person training were often provided to therapists to resolve potential technical issues at the outset (Acierno et al., 2016, 2017; Choi, Hegel, et al., 2014; Choi, Marti, et al., 2014; Goetter et al., 2014; Gros, Allan, et al., 2018; Gros, Lancaster, et al., 2018; Luxton et al., 2015, 2016; Yuen et al., 2013, 2015), and many studies arranged for technical support to be available as part of the study design (Acierno et al., 2016, 2017; Germain et al., 2009, 2010; Liu et al., 2019; Olden et al., 2017; Scogin et al., 2018; Watts et al., 2020; Yuen et al., 2013). Yuen et al. (2013, 2015) observed that technical difficulties reduced over the course of the study, in part due to participants becoming proficient at troubleshooting. Overall, while technical issues were encountered in most studies, participant feedback and reports from the study authors indicate that disruptions were not sufficiently impactful to detract from therapy.

#### Therapy relationship and process

On both formal measures and in qualitative reports, studies consistently reported that the videoconferencing clients were typically able to develop a positive connection with the therapist (Simpson et al., 2005, 2006; Simpson et al., 2015; Choi et al., 2013; Dunstan & Tooth, 2012; Fitt & Rees, 2012; Yuen et al., 2015), although some individual reports found a reduced sense of the therapist’s presence (e.g., Arnaet et al., 2007; Choi, Marti, et al., 2014). Furthermore, nearly all well‐powered RCTs that directly compared client ratings of the therapeutic relationship with in‐person delivery found no significant differences (Table 2), consistent with observations in smaller studies (e.g., Morgan et al., 2008; Scogin et al., 2018). Additionally, in an analysis of equivalence, Maieritsch et al. (2016) found confidence intervals for the working alliance fell within a priori bounds of equivalence in their trial of CPT. An exception to these findings is the CPT trial by Morland et al., (2015), which found statistically, but marginally, lower ratings for videoconferencing in the second session, with no differences at later time points. It is notable that, mirroring the adaptation to discomfort reported in some studies, some studies have also observed that videoconferencing clients rate a stronger alliance as sessions progress (Ertelt et al., 2011; Germain et al., 2010).

The converse finding of a stronger alliance in the videoconferencing condition by Watts et al. (2020) corresponds to qualitative comments in other studies to there being potential advantages of videoconferencing for the therapeutic relationship. Participants discussed finding therapy easier through having a greater sense of control (i.e., of emotion, of context, of the ability to leave) and the creation of a less intense therapy environment (Dunstan & Tooth, 2012; Fitt & Rees, 2012; Simpson, 2001; Simpson, Deans, & Brebner, 2001, Simpson et al. 2005; Simpson et al. 2006; Simpson et al. 2015). Participants discussed the ability to ‘talk more freely’, being less self‐conscious, finding it easier to communicate and feeling less pressured or intimidated in videoconferencing than they might be in‐person (Fitt & Rees, 2012; Simpson et al., 2005, 2015; Yuen et al., 2015).

Two of the three well‐powered studies that included both client and therapist ratings (Ertelt et al., 2011; Morland et al., 2015; Watts et al., 2020) identified differences of perspective: videoconferencing clients rated a stronger alliance than in‐person clients, while therapists rated the conditions the same (Watts et al., 2020), or clients rated the conditions similarly when therapists rated videoconferencing as inferior (Ertelt et al., 2011). These quantitative findings correspond to therapist reports of some difficulties in detecting emotion and ability to read body language through videoconferencing (Dunstan & Tooth, 2012; Simpson et al., 2005; Yu et al., 2020; Yuen et al., 2013). This highlights that therapists and clients may have discrepant experiences of videoconferencing therapy and that therapists can find the process of therapy more challenging, without that necessarily being reflected in client experience.

#### Adaptations of therapy

To deliver therapy via videoconferencing, several studies reported adaptations to therapy protocols. Most commonly, the practical logistic changes to how components of therapy were delivered involved using other technologies to share documents (e.g., mailing, faxing, emailing, or screen sharing worksheets and homework) (Gros et al., 2011; Himle et al., 2006; Lindsay et al., 2017; Luxton et al., 2015; Matsumoto et al., 2018; Turek, Yoder, Ruggiero, Gros, & Acierno, 2010). Clinical variations included removing situation‐specific in vivo exposure exercises from videoconferencing sessions and asking participants to complete exposure as homework only (Gros et al., 2011; Yuen et al., 2013), or creatively adapting or restricting exposure exercises so that they would be suitable for delivery within the virtual environment (e.g., talking on the phone to someone; Yuen et al., 2013). While feasible, for some clients, exposure tasks via videoconferencing were perceived to be less real and less engaging when compared with in‐person (Yuen et al., 2013). Conversely, where videoconferencing was delivered by smartphone, opportunities were identified in using the portability of the device to observe and conduct exposure activities within the person’s environment (Franklin et al., 2017; Turek et al., 2010; Vogel et al., 2012, Vogel et al., 2014).

Clients also reported that some activities translated less well such as meditation (Linsday et al., 2017) and use of imagery (Simpson et al., 2005) and that sensitive topics may be easier to discuss in person than over videoconferencing (Lindsay et al., 2017). It is notable that the studies surveyed used primarily behavioural models of treatment, so more reflective therapies are relatively less tested.

In addition to facilitating exposure tasks, the opportunity to see the person in their home environment was mentioned by some practitioners as helpful in contextualizing the person’s experiences (Lindsay et al., 2017) and potentially altering the power balance by seeing the person in their own territory (Simpson et al., 2005). However, environmental distractions arose more frequently within the home environment were also noted, and some clients treated the session less formally (e.g., smoking, attending wearing pyjamas), sometimes requiring boundary setting to maintain focus (Franklin et al., 2016; Lindsay et al., 2017; Yu et al., 2020). When contrasting with therapy delivered in‐person within the home, even greater distractions were noted when the therapist is in the home environment, and videoconferencing was noted to help to formalize the interaction and help clients stay focused (Choi, Hegel, et al., 2014).

## Discussion and conclusions

The literature to date shows consistent positive findings about the suitability of the videoconferencing modality for delivery of psychological therapies, with consistent findings that videoconferencing does not differ from in‐person therapy on outcome, satisfaction, therapy completion, and client experiences of the therapeutic alliance. Advantages include accessibility, particularly to persons without local in‐person services, but also in terms of convenience, reducing time and financial costs, and circumventing stigma, self‐consciousness and privacy concerns. Disadvantages include therapists finding it harder to judge body language, both clients and therapists experiencing initial discomfort with the modality while adapting to it, and interruptions arising from inevitable technical issues. However, it appeared that these concerns became less as clients and therapists adjusted: It may be that these issues become less of a concern as familiarity with videoconferencing grows across the population.

In considering the clinical populations for whom videoconferencing‐based therapy is most evidenced, conclusions primarily reflect the availability of trial data, rather than patterns of superior or inferior efficacy being observed. That withstanding, the videoconferencing modality has its most established evidence base in the delivery of cognitive behavioural therapies for PTSD and depression, where multiple trials have determined non‐inferiority to in‐person therapy. Across anxiety, obsessive–compulsive spectrum and eating disorders, there is also emergent evidence supporting use. Notably, there was a lack of study of videoconferencing therapy delivery to people with psychosis (although see Santestaban‐Echarri et al., 2020, for findings of broader service delivery, such as psychiatry appointments, being acceptable to this population).

Among therapy modalities, CPT, PE, and behavioural activation all have evidence of non‐inferiority, so can be considered the best supported for delivery in this modality. It is notable that trauma‐focused therapies, as some of the most confronting therapeutic approaches, are the best evidenced, which suggests that an in‐room presence is not required to deliver quite challenging therapies. Nonetheless, studies with available data have primarily utilized behavioural therapies. This leaves it unknown how therapies that use greater Socratic dialogue and reflection may operate within a videoconferencing environment, where some of the technical issues such as lag may prove more interruptive to the therapy process. Likewise, it is not known whether therapies for complex presentations, such as personality disorder and psychosis, which require more careful monitoring of in‐session rapport, would be equally successful.

A further caveat is that most large‐scale studies have been conducted in American veteran/military populations, with predominantly male participants, raising questions of generalizability. Of note, the clearest finding of videoconferencing having slightly poorer outcomes than in‐person therapy was in a trial for bulimia nervosa with a predominantly female group (although also using older telemedicine technology). On the other hand, the female‐only study by Morland et al. (2015) also found non‐inferiority and broadly similar results and observed that outcomes were in fact better among civilians compared with veterans. Nonetheless, these limitations in the available data urge some caution in assuming that videoconferencing therapies will function as well as in‐person therapy with all therapies and populations. It should also be noted that we did not aggregate and meta‐analyse mean scores for variables across studies, so there may be small between group differences that individual trials were not powered to detect.

In considering areas for further research, it is notable that since the COVID‐19 pandemic, new questions have emerged as in‐person therapy has not always been the most important reference point. We did not identify any studies that contrasted videoconferencing with telephone as the other widely accessible communication modality. While, in a non‐clinical client group, Day and Schnier (2002) failed to find any advantages of video counselling over telephone on process or outcome measures, considering the contrasting uses of these two widely accessible platforms, and understanding whether visual non‐verbal feedback facilitates maintaining rapport, is needed in clinical populations. Further study also needs to be directed at examining the opportunities that may be presented by using videoconferencing as a therapeutic medium. Observations suggest there are particular affordances of video, allowing access to the home environment and potentially for portability, which may have advantages in better connecting with the person’s daily life, and developing more ecologically valid therapeutic exercises. Rather than considering videoconferencing second to a presumed gold standard of in‐person therapy, attention to these affordances is needed to evolve videoconferencing‐based therapy practice in its own right.

## Conflict of interest

All authors declare no conflict of interest.

## Author contributions

Neil Thomas (Conceptualization; Data curation; Formal analysis; Investigation; Methodology; Supervision; Writing – original draft; Writing – review & editing) Caity McDonald (Conceptualization; Data curation; Formal analysis; Investigation; Writing – review & editing) Kathleen de Boer (Data curation; Formal analysis; Investigation; Writing – original draft; Writing – review & editing) Rachel M. Brand (Investigation; Writing – original draft; Writing – review & editing) Maja Nedeljkovic (Investigation; Writing – original draft; Writing – review & editing) Liz Seabrook (Conceptualization; Data curation; Formal analysis; Investigation; Writing – original draft; Writing – review & editing).

## Data Availability

The data that support the findings of this study are available from the corresponding author upon reasonable request.
